# Mechanical Degradation of Different Classes of Composite Resins Aged in Water, Air, and Oil

**DOI:** 10.1155/2019/7410759

**Published:** 2019-01-09

**Authors:** Weber Adad Ricci, Priscila Alfano, Saulo Pamato, Carlos Alberto dos Santos Cruz, Jefferson Ricardo Pereira

**Affiliations:** ^1^Department of Social Dentistry, Paulista State University, São Paulo, 04021-001, Brazil; ^2^Postgraduate Program of Health Sciences, University of Southern Santa Catarina, Tubarão, 88701-420, Brazil; ^3^Department of Dental Materials, Paulista State University, São Paulo, 04021-001, Brazil

## Abstract

A significant deterioration of the properties can drastically compromise the survival rate of restorative materials. The aim of this study was to assess flexural strength and hardness of three composite classes: hybrid composite resin (HCR), nanoparticulate composite resin (NCR), and silorane-based composite resin (SBCR). One hundred specimens were prepared for hardness testing by using a split metallic mold measuring 10 mm in diameter and 2 mm deep. Twenty specimens were prepared for each restorative material, randomly assigned for storage in air, distilled water, or mineral oil. After intervals of 24 hours, 30, 60, 90, and 120 days, hardness and flexural strength tests were initially compared in two levels: “storage medium” and “time” within each material group. A two-way analysis of variance was performed (p<0.05) on the variables “material” and “storage time” (p<0.05). The HCR showed to be stable with regard to the evaluation of flexural strength and hardness (p<0.05). A significant reduction occurs for the NCR in comparison to the other groups (p<0.05). The NCR presented the lowest values of hardness and flexural strength kept on water over time. The characteristics of material showed a strong influence on the decrease of the mechanical properties analyzed.

## 1. Introduction

Dental composites have been developed as an aesthetic alternative to the old amalgam-based restorative material and have become the most widely used materials in current dental practice. [1, 2] They became popular because of their ability to reproduce the natural color of teeth, are mercury-free, have low thermal conductivity, and adhere to tooth structure through adhesive systems. [3]

Despite the fact that technological advancements have encouraged the use of these materials in areas subjected to intense functional stress, physicochemical stability is necessary for these products to have acceptable longevity. Among the most significant factors contributing to the failure of composite resins over time are the loss of brightness, dental stains, marginal infiltration, recurrent decay, dental wear, and fractures. [4]

Industrial innovations such as nanotechnology and replacement of conventional monomers have promised to improve the mechanical quality of composite resins. Particle size changes and increased load in the composition, which would include the main physical properties of each resin class, such as maintaining nanoparticle polishing and resistance to wear and fracture in hybrid resins, have turned them into one of the most commonly studied materials.[5]

With regard to the monomers that make up the composite resins, one of the most recent studies within the field of dental materials was the introduction of silorane-based resins as a substitute for methacrylate-based resins. [6] Silorane-based resin is derived from oxirane and siloxane molecules that polymerize with cationic ring opening, overcoming clinical disadvantages of polymerization shrinkage of the well-established methacrylate-based materials. [7] Although the low shrinkage is not the principal factor that determines the superiority of a resin, silorane-based resin has shown good mechanical properties and stability in the oral cavity fluid simulators, encouraging its clinical use. [7–9]

A proper balance of mechanical properties should be established over time, given that a significant deterioration of the properties can drastically compromise the success rate of restorative materials. Because of the intense marketing by the dental industry, there is a fundamental necessity that in vitro and in vivo analyses clarify the mechanical and physical properties of technology-based nanoparticles in resin composite after storage for a long period. The aim of this study was to assess flexural strength and hardness of three composite classes: (a) hybrid, (b) nanoparticulate, and (c) silorane-based composites. The authors tested the null hypothesis that stated that there is no difference in flexural strength and hardness for the three composite classes, and behavior of the tested materials is similar for the three storage mediums at different storage times.

## 2. Materials and Methods

Several materials were used to carry out this study as shown in Table 1.

The experimental work was performed in accordance with the method described in ADA Specification No. 27 for resin-based filling composite materials, which is identical to ISO 4049:2009 (§7.12). [10]

One hundred specimens were prepared for hardness testing by using a split metallic mold measuring 10 mm in diameter and 2 mm deep. Masses of dental composites were compressed by using a glass microscope slide and a 1 kg metal disk with a hole in the center for placing the curing-light tip (Valo®, Ultradent, South Jordan, UT, USA) with a minimum light intensity of 500mW/cm^2^ measured by a radiometer (curing radiometer, Demetron/Kerr, Danbury, CT, USA). The polymerization time was 40 seconds through the glass slide and 40 seconds directly on the specimens. Twenty specimens were prepared for each restorative material (n=5), randomly assigned for storage in air, distilled water, or mineral oil (Nujol®, Schering-Plough, Rio de Janeiro, Brazil). The specimens remained in an incubator at 37±1°C throughout the experiment.

Readings were made after periods of 24 hours, 30, 60, 90, and 120 days by using a durometer (Buehler, Lake Bluff, USA) equipped with a Vickers diamond and a compression load of 100 gf applied for 30 seconds. Measurements were obtained through two dents per quadrant, totaling eight readings for each specimen.

A split metallic stainless steel bar matrix 12 mm long, 2 mm wide, and 1 mm thick was used to prepare the samples for flexural strength testing (n=450). It was embedded into a rectangular aluminum metallic base. The composite resin materials were applied into the root cavity and compressed by using a glass microscope slide and a metal disk with a mass of 1 kg, which had a hole in the center for placing the curing-light tip (Valo®, Ultradent, South Jordan, UT, USA) with a minimum light intensity of 500mW/cm^2^ measured by a radiometer (curing radiometer, Demetron/Kerr, Danbury, CT, USA). Polymerization time was 120 seconds, with 40 seconds for each segment of approximately 4 mm long. One hundred and fifty specimens of each material were prepared (n=10) and were randomly assigned for storage in air, distilled water, or mineral oil (Nujol®, Schering-Plough, Rio de Janeiro, Brazil). After intervals of 24 hours, 30, 60, 90, and 120 days, 10 specimens were randomly selected for flexural strength testing. Throughout the experiment, all specimens remained in the respective storage mediums in light-protected containers and were incubated at 37±1°C.

For the three-point-bend strength test, two metal devices were used, composed of a table with two cylindrical support sections of 1.6 mm in diameter, 10 mm apart from each other, and a beam or rod for the application of compressive stress in the center of the upper face of the specimen, with an active tip cylindrical section, 3.2 mm in diameter. Assays were performed by using an MTS 810 machine (MTS Systems Corp., Eden Prairie, MN, USA) with 10 kN load cell and a crosshead speed of 0.5 mm/min. The following equation was used to calculate flexural strength values: RF=3LF/2bh^2^, where

- RF is the flexural strength, (MPa);
- L is the span length between the supports, (mm);
- F is the applied load at fracture, (N);
- b is the width of the specimen, (mm);
- h is the thickness of the specimen, (mm).

Hardness and flexural strength tests were initially compared in two levels, “storage medium” and “time” within each material group.

For power analysis, sample size was calculated on the basis of previous sample size calculations performed in similarly designed studies. Based on previous investigations, a power analysis determined that, for an alpha value of 5% and a power of 80%, a sample size of 10 specimens per group would be required. A two-way analysis of variance was performed (p<0.05). Comparisons among the restorative materials were subsequently made for each mechanical property, only in the early periods, prior to immersion, and at the end of the process, after 120 days, in distilled water and mineral oil. New two-way analyses of variance were performed on the variables “material” and “storage time” (p<0.05). Additional Tukey tests were performed in all analyses, with the same level of significance used throughout the experiment (p<0.05).

## 3. Results and Discussion

Table 2 shows the significant variability for the “storage medium” factor and reinforces the stability for the “time” factor. The hardness test for the HCR showed an adverse action of mineral oil, with a reduction around 29%, identified soon after the immersion.

Table 3 shows a nonsignificant variability for the factor “storage medium” (p>0.05). However, it does not emphasize the detected resistance loss after 30 days of storage. Anyway, in this table, a decrease around 18% can be observed for the HCR in the three storage mediums after the initial evaluation.

Table 4 shows the significant variability for the factors “storage medium” and “time” (p<0.05). Whereas the reduction observed after immersion in distilled water was around 13%, with stabilization at 30 days, hardness reduction provided by mineral oil was about 18% and was soon detected after immersion. The average percentage reduction provided by time, with stabilization at 30 days, was around 8%. With regard to NCR, hardness reduction percentage, even without immersion, in distilled water and mineral oil was around 5% and 10%, respectively.

Table 5 shows the strength loss observed in an isolated analysis of the factors under study and reveals the difference in behavior among the storage mediums evaluated (p<0.05). Reduction caused only by specimen aging stored in air was around 43%, with stabilization after 30 days. When stored in water, the total reduction was around 72%, with stabilization after 60 days of storage. When mineral oil was stored, contrary to that observed in an isolated analysis, there was a reduction, but, compared with the value observed prior to immersion, it was less severe, around 46%, with stabilization after 60 days of immersion. The reduction by the sole action of water was approximately 29%, whereas the reduction by the sole action of mineral oil was approximately 3%.

Table 6 shows a significant variability related to the “storage medium” factor of the SBCR (p<0.05). Whereas no hardness reduction occurred when stored in air and water, lower values around 22% were observed when stored in mineral oil, after 24 hours of immersion.

Table 7 shows a significant variability observed for immersion in distilled water (p<0.05). However, it does not emphasize the detected resistance loss, in an isolated analysis, after immersion in mineral oil. Similarly, it does not emphasize the changes in the “time” factor. Anyway, this table shows that the initial tendency to increase the flexural strength of SBCR, in the three storage mediums, is reduced after 30 days. However, when the material is stored in air, there is an initial increase of approximately 18% and a subsequent reduction of approximately 10%. When stored in mineral oil and water, the behavior is inverted, and there is a greater reduction provided by immersion. When stored in water, the initial increase of about 25% is totally compromised by a reduction of approximately 35%. When stored in mineral oil, the initial increase of approximately 9% is overcome by a reduction of 13%. Considering as maximum reference the value of 72.5 MPa, obtained in dry conditions 30 days after storage, and discounting the loss of about 10% registered even without immersion, the reduction of flexural strength provided by water and mineral oil was around 20% and 14%, respectively.

A comparison between the composite resins was made by using the Tukey test (p<0.05).  Table 8 shows that all materials tested suffered detrimental changes in hardness related to the storage time factor. With regard to the materials stored in water, NCR and SBCR had greater reduction rates compared with other materials (p<0.05). The HCR presented the greatest hardness stability value when stored in water.

The HCR group showed to be stable with regard to the “time/storage medium” factor in the evaluation of flexural strength (Table 9). A significant reduction occurs for the NCR group in comparison to the other groups (p<0.05). The characteristic of this material showed a strong influence on the decrease of the mechanical properties analyzed.

The results from this study rejected the null hypothesis in relation to the type of material and storage medium. The different types of material demonstrated significant action on the hardness and flexural strength values in relation to the storage times and storage mediums studied (p<0.05). Previous works have demonstrated deterioration of composite resins when immersed in different mediums. [11, 12] The most commonly tested storage mediums were water, oil, and alcohol. However, in in vivo experiments, other factors may act to stimulate and even accelerate the deterioration process, turning it critical. Thus, negative results obtained in vitro in pure mediums may be enhanced at critical levels when these materials are used in the mouth. The storage mediums used in the experiment, besides the control group stored in air, represented the conditions commonly found in the oral cavity, such as saliva, beverages, medicines, and fatty foods. [13] They have shown to be able to adversely affect the mechanical properties of investigated products.

In order to promote common challenges in the oral environment, different immersion was used, especially in the possibility of promoting an antagonistic action between an aqueous medium (water) and a lipoic environment, as suggested in previous studies. [13]

The adverse effect of water is particularly reported by several authors. [14, 15] Initially, there may be a softening of the organic matrix by hygroscopic expansion, by solubilizing unsaturated monomers or by breakage of macromolecule chemical bonds, resulting in higher rates of boundary slip between the polymer chains. [16–18] In a next step, hydrolysis or hydration of siloxane bonds of the silane layer will occur due to the degradation of matrix-filler bonds; surface or internal cracks and porosity would facilitate water access to this interface. [13, 19] Finally, there could be solubilization of the particles by releasing ions of the components [20, 21] and the presence of other solvents, lubricants, electrolytes, or enzymes, [15, 21, 22] along with mechanical cycling that would accelerate the process, occurs. According to this analysis, silane plays an important role as a binding agent in the chemical and mechanical stability of these materials. Once this connection is compromised, the material becomes fragile. Of all the classes of materials studied, SBCR group was the least affected by water given that, with 60% of small particles of quartz and new silorane-based matrix, which is less hydrophilic, it was only affected by mineral oil. It had a reduction of 24% and stabilized in 24 hours, with overall stability in the remaining immersion mediums, after 60 days, although with lower relative hardness. However, flexural strength testing of water activity on bonding agent might explain the resistance reduction of this product. [7]

The NCR group, at 60% compressive load, composed of silica agglomerates/zirconia and dimethacrylate-based matrices (Bis-GMA, UDMA, Bis-EMA, and TEGDMA), proved to be affected by all three storage mediums: air (5% reduction and stabilization in 7 days), water (14% reduction and stabilization in 7 days), and mineral oil (16% reduction and stabilization in 24 hours). However, the HCR group, at 60% compression load, composed of silica/zirconia and dimethacrylate-based matrices (Bis-GMA, UDMA, Bis-EMA, and TEGDMA), proved to be affected only by the mineral oil and with the same degree of reduction (16%) and stabilization time (24 hours). Assuming the same monomer combination for these two materials, one can conclude that, somehow, the nanoclusters of NCR material have resulted in hardness reduction, even in dry conditions, without immersion.

In the flexural strength tests, the authors observed slightly different order from that obtained in the hardness test. Prior to storage, there was a statistical superiority for HCR group (94.2 MPa) and NCR (105.6 MPa), both dimethacrylate-based matrices. However, they had different volume content compressive strengths of 71% and 60%, respectively. The SBCR filled with fine quartz particles (61.2 MPa), even with filler content similar to that of NCR, had inferior mechanical properties. It should be noted that mechanical properties are not dependent exclusively from the filler content (inorganic phase) but an association with the monomer constitution (organic phase).

In the flexural strength tests, tensile stress is responsible for specimen fracture. Thus, in addition to cohesion of the organic phase (composition and maximum degree of conversion), the effective bonding between the matrix and filler particles provided by the silane-based agent influences the outcome. The composite amount and the total interface area, favoring propagation, might be responsible for the observed differences. Nanoparticle agglomerates used as a filler in NCR, even in a small amount, may have provided greater flexural strength. However, this composite showed significant deterioration of flexural strength after storage in water during the tested period. Considering that flexural strength of the HCR was not affected by water, a greater sensitivity to hydrolytic degradation of the silane interface can be assumed, because of the increased surface area provided by the nanoparticles, the main constituents of nanoclusters, used as filler in this material. Flexural strength of the silorane-based matrix of the SBCR group may have been affected by solubilization of monomers partially insaturated or by the breaking of macromolecules.

The tests of materials stored in air medium showed flexural strength losses of around 18% for the HCR, 43% for the NCR, and 10% for the SBCR group. According to Bijelic-Donova et al. [21], volatilization of double unsaturated monomer components may explain the resistance losses even without immersion.

Limitations of this study include the in vitro conditions, which could not completely replicate clinical conditions. Further studies should incorporate thermocycling to obtain more meaningful results. Clinical studies are important to confirm the present study results, as well as the results found by others in in vitro investigations.

## 4. Conclusions

Considering the limitations of this study, it is possible to conclude that the characteristics of material showed a strong influence on the decrease of the mechanical properties analyzed. Then, the mechanical tests indicated difference in flexural strength and hardness for the three composite classes, and behavior of the tested materials is similar for the three storage mediums at different storage times. The NCR group presented the lowest values of hardness and flexural strength kept on water over time.

## Figures and Tables

**Table 1:** Composite resin systems investigated in this study.

Composite / manufacturer	Composition of the organic matrix*∗*	Inorganic phase composition*∗*	Color	Manufacturing lot number	commercial classification
Filtek Z-100 (3M Dental Products, St. Paul, MN, USA)	Bis-GMA, TEGDMA	zirconia/silica 4.5 *μ*m (maximum size)	A 3.5	7EP	Hybrid
		71% by volume			
Filtek Z-350 (3M Dental Products, St. Paul, MN, USA)	Bis-GMA, UDMA Bis-EMA, TEGDMA	zirconia/silica 0,005-0,02 *μ*m (5-20 nm) 0,6-1,4 *μ*m (agglomerate)	A 3.5	9AK	Nanocomposite
		59.5% by volume			
Filtek P-90 (3M Dental Products, St. Paul, MN, USA)	Silorane	quartz 0,4 um (medium size)	A 3.5	N130928	Silorane-based
		58%, by volume			

Organic matrix composition and compressive load were provided by the manufacturer.

**Table 2:** Mean, standard error, and critical value as measured by the Tukey test for hardness values of the interaction between “storage medium” and “time” of HCR group (VHN).

	24 hours	30 days	60 days	90 days	120 days
AIR	86.6 (a) A	80.9 (a) A	80.6 (a) A	82.2 (a) A	82.1 (a) A
WATER	82.7 (a) A	80.7 (a) A	79.1 (a) A	79.0 (a) A	79.0 (a) A
MINERAL OIL	59.0 (a) B	58.3 (a) B	57.5 (a) B	57.4 (a) B	57.3 (a) B
Standard error = 2.00					

Critical value of 5% = 10.0. Horizontal, lowercase letters (same material, different storage conditions). Vertical, capital letters (same storage condition, different materials).

**Table 3:** Mean, standard error, and critical value as measured by the Tukey test for the flexural strength values of the interaction between “storage medium” and “time” of HCR group (MPa).

	24 hours	30 days	60 days	90 days	120 days
AIR	94.2 (a) A	75.1 (a) A	73.7 (a) A	72.0 (a) A	70.7 (a) A
WATER	90.4 (a) A	77.8 (a) A	74.2 (a) A	72.6 (a) A	71.5 (a) A
MINERAL OIL	86.3 (a) A	77.6 (a) A	74.7 (a) A	74.1 (a) A	71.4 (a) A
standard error = 5,67					

Critical value of 5% = 27.51. Horizontal, lowercase letters (same material, different storage conditions). Vertical, capital letters (same storage condition, different materials).

**Table 4:** Mean, standard error, and critical value as measured by the Tukey test for hardness values of the interaction between “storage medium” and “time” of NCR group (VHN).

	24 hours	30 days	60 days	90 days	120 days
AIR	66.7 (a) A	62.2 (a) A	61.0 (a) A	61.8 (a) A	61.8 (a) A
WATER	68.2 (a) A	59.1 (b) A	59.2 (b) A	58.3 (b) AB	58.3 (b) AB
MINERAL OIL	55.7 (a) B	54.9 (a) A	53.8 (a) A	53.9 (a) B	53.8 (a) B
standard error = 1.48					

Critical value of 5% = 7.42. Horizontal, lowercase letters (same material, different storage conditions). Vertical, capital letters (same storage condition, different materials).

**Table 5:** Mean, standard error, and critical value as measured by the Tukey test for flexural strength values of the interaction between “storage medium” and “time” of NCR group (MPa).

	24 hours	30 days	60 days	90 days	120 days
AIR	105.6 (a) A	59.3 (b) A	58.6 (b) A	58.0 (b) A	57.1 (b) A
WATER	71.6 (a) B	63.1 (a) A	29.5 (b) B	29.4 (b) B	28.9 (b) B
MINERAL WATER	82.9 (a) AB	68.5 (ab) A	57.6 (b) A	58.3 (b) A	56.4 (b) A
standard error = 4.80					

Critical value of 5% = 23.29. Horizontal, lowercase letters (same material, different storage conditions). Vertical, capital letters (same storage condition, different materials).

**Table 6:** Mean, standard error, and critical value as measured by the Tukey test for hardness values of the interaction between “storage medium” and “time” of SBCR group (VHN).

	24 hours	30 days	60 days	90 days	120 days
AIR	47.1 (a) A	45.5 (a) A	45.3 (a) A	45.6 (a) A	45.6 (a) A
WATER	44.2 (a) AB	43.8 (a) AB	46.9 (a) A	45.7 (a) A	45.6 (a) A
MINERAL OIL	35.9 (a) B	35.3 (a) B	34.8 (a) B	34.9 (a) B	34.7 (a) B
standard error = 1.79					

Critical value of 5% = 8.94. Horizontal, lowercase letters (same material, different storage conditions). Vertical, capital letters (same storage condition, different materials).

**Table 7:** Mean, standard error, and critical value as measured by the Tukey test for flexural strength values of the interaction between “storage medium” and “time” of SBCR group (MPa).

	24 hours	30 days	60 days	90 days	120 days
AIR	61.2 (a) A	72.5 (a) A	66.2 (a) A	65.5 (a) A	64.5 (a) A
WATER	62.6 (a) A	78.1 (a) A	52.4 (b) A	50.8 (b) A	49.9 (b) A
MINERAL OIL	58.1 (a) A	63.3 (a) A	56.5 (a) A	55.1 (a) A	54.4 (a) A
standard error = 4.24					

Critical value of 5% = 20.55. Horizontal, lowercase letters (same material, different storage conditions). Vertical, capital letters (same storage condition, different materials).

**Table 8:** Mean, standard error, and critical value as measured by the Tukey test for hardness values of the interaction between “type of material” and “time/storage medium” (VHN).

	BASELINE AIR	120 DAYS DISTILLED WATER	120 DAYS MINERAL OIL
HCR	86.6 (a) (A)	79.0 (a) (A)	57.3 (b) (A)
NCR	66.7 (a) (B)	58.3 (b) (B)	53.8 (b) (A)
SBCR	47.2 (a) (C)	45.6 (a) (C)	34.7 (b) (B)
standard error = 1.879			

Critical value of 5% = 8.198. Horizontal, lowercase letters (same material, different storage conditions). Vertical, capital letters (same storage condition, different materials).

**Table 9:** Mean, standard error, and critical value as measured by the Tukey test for flexural strength values of the interaction between “type of material” and “time/storage medium” (MPa).

	BASELINE AIR	120 DAYS DISTILLED WATER	120 DAYS MINERAL OIL
HCR	94.2 (a) (A)	71.5 (a) (A)	71.4 (a) (A)
NCR	105.6 (a) (A)	28.9 (b) (B)	56.4 (b) (A)
SBCR	61.2 (a) (B)	49.9 (a) (AB)	54.4 (a) (A)
Standard error = 8,50			

Critical value of 5% = 38.33. Horizontal, lowercase letters (same material, different storage conditions). Vertical, capital letters (same storage condition, different materials).

## Data Availability

The data used to support the findings of this study are available from the corresponding author upon request.

## References

[1] Randolph L. D., Palin W. M., Leloup G., Leprince J. G. (2016). Filler characteristics of modern dental resin composites and their influence on physico-mechanical properties. *Dental Materials*.

[2] Sideridou I. D., Vouvoudi E. C., Keridou I. V. (2015). Sorption characteristics of oral/food simulating liquids by the dental light-cured nanohybrid composite Kalore GC. *Dental Materials*.

[3] Cazzaniga G., Ottobelli M., Ionescu A., Garcia-Godoy F., Brambilla E. (2015). Surface properties of resin-based composite materials and biofilm formation: A review of the current literature. *American Journal of Dentistry*.

[4] Kumar N., Shortall A. (2014). Performance of the experimental resins and dental nanocomposites at varying deformation rates. *Journal of Investigative and Clinical Dentistry*.

[5] Saen P., Atai M., Nodehi A., Solhi L. (2016). Physical characterization of unfilled and nanofilled dental resins: Static versus dynamic mechanical properties. *Dental Materials*.

[6] McHugh L. E. J., Politi I., Al-Fodeh R. S., Fleming G. J. P. (2017). Implications of resin-based composite (RBC) restoration on cuspal deflection and microleakage score in molar teeth: Placement protocol and restorative material. *Dental Materials*.

[7] Magno M. B., Nascimento G. C. R., da Rocha Y. S. P., d'Paula Gonçalves Ribeiro B., Loretto S. C., Maia L. C. (2016). Silorane-based composite resin restorations are not better than conventional composites - A meta-analysis of clinical studies. *The Journal of Adhesive Dentistry*.

[8] Barceleiro M. O., Soares G. M., Espindola O., Kahn S., Poiate I. A. V. P., Filho H. R. S. (2015). Low-shrinkage composites: An in vitro evaluation of sealing ability after occlusal loading. *General dentistry*.

[9] Santos M. J. M. C., Rêgo H. M. C., Mukhopadhyay A., Najjar M. E., Santos G. C. (2016). Effect of artificial aging on the surface roughness and microhardness of resin-based materials. *General dentistry*.

[10] International Standard ISO 4049 Dentistry - polymer-based restorative materials, 2009.

[11] Gezawi M. E., Kaisarly D., Al-Saleh H., ArRejaie A., Al-Harbi F., Kunzelmann K. H. (2016). Degradation potential of bulk versus incrementally applied and indirect composites: Color, microhardness, and surface deterioration. *Operative Dentistry*.

[12] Da Silva S., Da Silva E. M., Delphim M. B. F., Poskus L. T., Amaral C. M. (2015). Influence of organic acids present in oral biofilm on the durability of the repair bond strength, sorption and solubility of resin composites. *American Journal of Dentistry*.

[13] Krüger J., Maletz R., Ottl P., Warkentin M., Mishra Y. K. (2018). In vitro aging behavior of dental composites considering the influence of filler content, storage media and incubation time. *PLoS ONE*.

[14] de Assis F. S., Lima S. N. L., Tonetto M. R. (2016). Evaluation of bond strength, marginal integrity, and fracture strength of bulk- vs incrementally-filled restorations. *The Journal of Adhesive Dentistry*.

[15] Krämer N., Reinelt C., Frankenberger R. (2015). Ten-year clinical performance of posterior resin composite restorations. *The Journal of Adhesive Dentistry*.

[16] Wang X., Huyang G., Palagummi S. V. (2017). High performance dental resin composites with hydrolytically stable monomers. *Dental Materials*.

[17] Huang B., Siqueira W. L., Cvitkovitch D. G., Finer Y. (2018). Esterase from a cariogenic bacterium hydrolyzes dental resins. *Acta Biomaterialia*.

[18] Van De Sande F. H., Da Rosa Rodolpho P. A., Basso G. R. (2015). 18-year survival of posterior composite resin restorations with and without glass ionomer cement as base. *Dental Materials*.

[19] Panahandeh N., Torabzadeh H., Naderi H., Sheikh-Al-Eslamian S. M. (2017). Effect of water storage on flexural strength of silorane and methacrylate-based composite resins. *Restorative Dentistry & Endodontics*.

[20] Manojlovic D., Dramićanin M. D., Milosevic M. (2016). Effects of a low-shrinkage methacrylate monomer and monoacylphosphine oxide photoinitiator on curing efficiency and mechanical properties of experimental resin-based composites. *Materials Science and Engineering C: Materials for Biological Applications*.

[21] Bijelic-Donova J., Garoushi S., Lassila L. V. J., Vallittu P. K. (2015). Oxygen inhibition layer of composite resins: effects of layer thickness and surface layer treatment on the interlayer bond strength. *European Journal of Oral Sciences*.

[22] Sidi A., Larché J.-F., Bussière P.-O., Gardette J.-L., Therias S., Baba M. (2015). Water as a morphological probe to study polymer-filler interfaces: An original application of thermoporosimetry. *Physical Chemistry Chemical Physics*.

